# Targeting the ROS-JNK/p38 Axis: Schisandrin A as a Novel Therapeutic Candidate for Esophageal Squamous Cell Carcinoma

**DOI:** 10.4014/jmb.2603.03024

**Published:** 2026-04-17

**Authors:** Minjun Lee, Sang Hoon Joo, Yung Hyun Choi, Goo Yoon, Jin Woo Park, Jung-Hyun Shim

**Affiliations:** 1 Department of Biomedicine, Health & Life Convergence Sciences, BK21 Four, College of Pharmacy, Mokpo National University, Muan 58554, Republic of Korea; 2 College of Pharmacy, Daegu Catholic University, Gyeongsan 38430, Republic of Korea; 3 Department of Biochemistry, College of Korean Medicine, Dong-Eui University, Busan 47227, Republic of Korea; 4 Department of Pharmacy, College of Pharmacy, Mokpo National University, Muan 58554, Republic of Korea; 5 The China-US (Henan) Hormel Cancer Institute, Zhengzhou, Henan 450008, P.R. China

**Keywords:** Schisandrin A, Esophageal squamous cell carcinoma, Reactive oxygen species, Apoptosis, JNK, p38

## Abstract

Esophageal squamous cell carcinoma (ESCC) is a highly lethal malignancy with limited therapeutic options. Schisandrin A (Sch A), a bioactive lignan, demonstrates anti-cancer potential, but its effectiveness against ESCC has yet to be investigated. Human ESCC cell lines (KYSE30 and KYSE510) and normal HEKa cells were treated with Sch A at concentrations ranging from 10 to 80 μM. We assessed cell viability, colony formation, apoptosis, ROS production, mitochondrial membrane potential, and cell cycle distribution. The expression of proteins involved in JNK/p38 MAPK signaling, Bcl-2 family members, and cell cycle regulators was analyzed using Western blotting. Specific inhibitors (SP600125, SB203580, NAC, Z-vad-fmk) were employed to validate the underlying mechanisms. Sch A reduced cell viability and colony formation in KYSE30 and KYSE510 cells in a dose-dependent manner while sparing normal HEKa cells. It induced apoptosis, G0/G1 phase arrest, ROS generation, and caspase activation. Notably, these effects were partially reversed by pathway-specific inhibitors. Sch A activated JNK/p38 MAPK signaling, downregulated Bcl-2 and Mcl-1, upregulated Bax and Bad, and modulated cell cycle regulators such as cyclin D1, CDK4/6, and p27. Sch A selectively induces apoptosis in ESCC cells through ROS-JNK/p38-mediated pathways, mitochondrial dysfunction, and cell cycle arrest. These findings indicate that Sch A is a promising therapeutic candidate for treating ESCC.

## Introduction

Esophageal squamous cell carcinoma (ESCC) continues to pose a significant health burden globally, claiming over four hundred thousand lives each year [1]. Despite recent advances in cancer therapeutics, the prognosis for patients with advanced ESCC remains discouraging, with 5-year survival rates falling below 20% in many regions [2]. Although systemic chemotherapy for ESCC, based on 5-fluorouracil and cisplatin [3], has been redefined with the integration of immune checkpoint inhibitors [4], many patients still do not respond [5], and severe treatment-related toxicities often result in therapy discontinuation [6].

Resistance to anticancer therapeutics and immunotherapy, whether intrinsic or acquired, limits long-term clinical benefits. The low response rates and significant toxicities associated with current chemotherapeutic regimens for ESCC, coupled with the biological heterogeneity of the disease, highlight the urgent need for alternative therapeutics that offer improved efficacy and reduced side effects.

Natural products derived from medicinal plants have been investigated as potential anticancer agents due to their ability to simultaneously modulate multiple oncogenic pathways while often maintaining favorable safety profiles [7]. Quercetin, a flavonoid compound found in vegetables and fruits, has potential anticancer activity [8]. Crocetin from saffron reportedly inhibits the proliferation of ESCC cells by inducing apoptosis [9].

Schisandrin A (Sch A), a bioactive dibenzocyclooctadiene lignan isolated from *Schisandria chinensis* [10], exhibits potent antitumor activity against various malignancies, including colorectal [10], breast [11], and prostate [12] cancers. In colorectal cancer cells, Sch A inhibits tumor cell growth and survival by suppressing heat shock factor 1-dependent transcriptional activation [13].

In prostate cancer cells, Sch A induces apoptosis by generating reactive oxygen species (ROS), which leads to endoplasmic reticulum (ER) stress and the subsequent activation of JNK MAPK signaling [12]. These findings suggest that Sch A may exert antitumor effects by regulating stress response and survival signaling pathways in ESCC cells. Although the anticancer activity of Sch A alone has not been extensively studied, a microemulsion containing docetaxel and Sch A has been evaluated for its therapeutic effects in esophageal carcinoma [14]. The combination of docetaxel and Sch A in the microemulsion enhanced drug accumulation in tumor cells, helping to overcome multidrug resistance and demonstrating superior tumor growth inhibition compared to docetaxel alone. This suggests that Sch A may function as both a cytotoxic compound and a chemosensitizer. In this study, we investigated the anticancer activity of Sch A in human ESCC cell lines KYSE30, KYSE510, KYSE70, KYSE410, and KYSE450 to determine its therapeutic potential as a standalone or adjunctive agent in treating ESCC.

## Materials and Methods

### Reagents and Antibodies

Schisandrin A (MW 416.51, 98% purity), Z-vad-fmk, SB203580, SP600125, N-acetylcysteine (NAC), oxaliplatin (Ox), and Basal Medium Eagle (BME) were obtained from Sigma-Aldrich (USA). 0.05% trypsin, fetal bovine serum (FBS), penicillin-streptomycin (p/s), and phosphate buffered saline (PBS) were acquired from Gibco (USA). RPMI 1640 were obtained from WELGENE (Republic of Korea). 3-(4,5-dimethyl-2-thiazolyl)-2,5-diphenyl-2-H-tetrazolium bromide (MTT) were purchased from Biosesang (Republic of Korea). Primary antibodies against Mcl-1 (sc-12756), Bcl-xL (sc-8392), Bcl-2 (sc-7382), BID (sc-56025), Bad (sc-8044), Apaf-1 (sc-33870), caspase-3 (sc-7148), cyclin D1 (sc-20044), Cdk4 (sc-70831), Cdk6 (sc-7961), p27 (sc-56338), and β-actin (sc-47778) were sourced from Santa Cruz Biotechnology (USA). Primary antibodies targeting p38 (#9212) and p-p38 (Thr180/Tyr182) (#9211), Bax (#5023), PARP (#9542), JNK (#9252), p-JNK (Thr183/Tyr185) (#4668) were procured from Cell Signaling Biotechnology (USA).

### Cell Culture and Treatment

The human ESCC cell lines KYSE30, KYSE70, KYSE410, KYSE450, and KYSE510 were procured from the Chinese Academy of Sciences (China) and were maintained in RPMI 1640 with additional supplements of 1% penicillin/streptomycin, 10% FBS. Human immortalized keratinocyte cell line (HEKa) was procured from the American Type Culture Collection (USA) and were incubated in DMEM with additional supplements of 1% penicillin/streptomycin, 10% FBS. Cells were maintained at 37°C in a 5% CO_2_ incubator. The KYSE30 and KYSE510 cells were treated using each concentration of NAC (5 mM), SP600125 (6 μM), SB203580 (10 μM), or Z-vad-fmk (30 μM) for 3 h, and then exposed to 60 μM Schisandrin A for 48 h.

### Cell Viability Assay

KYSE30 cells (2.75 × 10^3^ cells/well), KYSE70 cells (12.3 × 10^3^ cells/well), KYSE410 cells (4 × 10^3^ cells/well), KYSE450 cells (4.5 × 10^3^ cells/well), KYSE510 cells (4.75 × 10^3^ cells/well), HEKa cells (8 × 10^3^ cells/well) were plated in 96-well microtiter plates overnight, treated with Schisandrin A for 24 h or 48 h. MTT reactions were performed for 1h before addition of formazan solvents (DMSO).The supernatant was subsequently aspirated, and 100 μl of DMSO was dispensed to each well. Absorbance was determined at 570 nm on a microplate reader (Thermo Fisher Scientific, USA).

### Soft Agar Assay

Briefly, KYSE30 (8 × 10^3^ cells/well) and KYSE510 (8 × 10^3^ cells/well) were cultured in 0.3% agar containing Schisandrin A or Ox, and after 14 days of culture at 37°C with 5% CO_2_. The colonies were observed using a microscope (Leica Microsystems, Germany), and counts and areas were determined using a colony plug of integrated i-Solution (Canada).

### Annexin V-FITC/PI Double Staining Assay

KYSE30 cells (2.75 × 10^3^ cells/well) and KYSE510 cells (4.75 × 10^3^ cells/well) were plated in 60 mm plates and exposed to various concentrations of Schisandrin A for 48 h. Annexin V-FITC (Miltenyi Biotec) and PI were introduced into cell suspensions and additionally incubated for 20 min under light-protected conditions. Cellular fluorescence was analyzed by MACSQuant 16 Analyzer as described above.

### Western Blotting

Cells were lysed with RIPA buffer (iNtRON Biotechnology, Republic of Korea), and the collected protein concentration was quantified with a BCA assay kit (Bio-Rad, USA) method. Equal amounts of protein were resolved by SDS–PAGE and transferred onto PVDF membranes. After membrane blocking, the membranes were probed using primary antibodies for 12–16 h at 4°C, followed by incubation with HRP-conjugated secondary antibodies. Chemiluminescence signals were collected and quantified using an iBright CL1500 Imaging System (Thermo Fisher Scientific).

### Analysis of Reactive Oxygen Species (ROS)

Intracellular ROS levels were assessed by staining cells with 5 μM CellROX Green reagent (Invitrogen, USA). Then, the cells were washed with HBSS, and the MACSQuant 16 Analyzer.

### Cell Cycle Analysis

Briefly, for cell cycle determination, equal numbers of KYSE30 cells (2.75 × 10^3^ cells/well), KYSE510 cells (4.75 × 10^3^ cells/well) were plated in 60 mm plate per well and incubated with different concentration of Schisandrin A (0, 20, 40, 60 μM) or Ox (10 μM) for 48 h. The cells were preserved gently through the addition of 70% ethanol before they were placed in a –20°C freezer for 12–16 h. Cells were rinsed with PBS and stained with PI (5 μl) and RNase A (2.5 μl) under light-protected conditions for 20 min. Cell cycle distribution data were acquired using a MACSQuant Analyzer 16 (Miltenyi Biotec, Germany).

### JC-1 Staining

The KYSE30 cells and KYSE510 cells seeded in 60mm plates were subjected to Schisandrin A (0, 20, 40, and 60 μM), Ox (10 μM) treatments for 48 h. The cells were detached and suspended in RPMI-1640 medium containing JC-1 staining buffer at 37°C for 10 min. The fluorescence intensity ratios (red/green) were subsequently analyzed by MACSQuant VYB Analyzer.

### MultiCaspase Assay

The activation of caspases was analyzed using the Muse MultiCaspase Assay Kit (MCH100109, Merck Millipore), followed by analysis on a Muse Cell Analyzer (Merck Millipore).

### Statistical Analysis

All statistical analyses were carried out with the KaleidaGraph version 4.5 (Synergy Software, USA) test. Differences among multiple groups were assessed using one-way analysis of variance (ANOVA), with Tukey’s post hoc multiple comparison analysis. Statistical significance was set at * *p* < 0.05, ** *p* < 0.01, and *** *p* < 0.001.

## Results

### Sch A Exerts Cytotoxicity in ESCC Cells

**Fig. 1:**
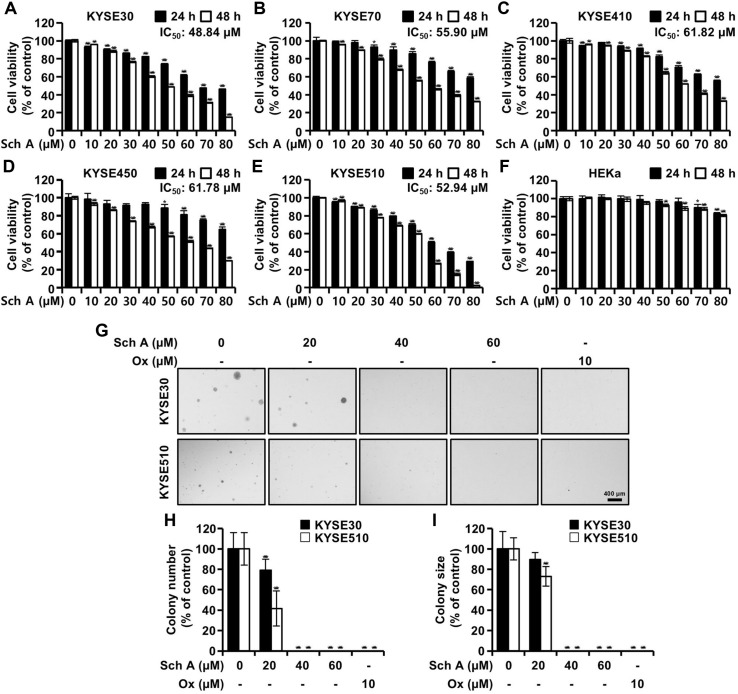
Cell viability and growth. (**A–F**) Human ESCC cell lines and HEKa cells were treated with varying concentrations of schisandrin A (Sch A; 10–80 μM) for 24 h (closed bars) or 48 h (open bars), and cell viability was measured using the MTT assay. (**A**) Cell viability of KYSE30 cells following Sch A treatment. (**B**) Cell viability of KYSE70 cells. (**C**) Cell viability of KYSE410 cells. (**D**) Cell viability of KYSE450 cells. (**E**) Cell viability of KYSE510 cells. (**F**) Cell viability of HEKa cells. IC_50_ values are for 48 h incubation. Data are shown as mean ± standard deviation (n = 9). *p < 0.05, **p < 0.01, and ***p < 0.001 versus blank. (**G–I**) KYSE30 and KYSE510 cells were exposed to the indicated concentrations of schisandrin A (Sch A; 0, 20, 40, and 60 μM) or oxaliplatin (Ox; 10 μM) and grown on soft agar plates. (**G**) Representative microscopic images. (**H, I**) The number and size of colonies were quantified using i-Solution software. (**H**) Number of colonies relative to the blank. (**I**) Colony size relative to the blank. Data are presented as mean ± standard deviation (n = 3). ***p < 0.001 versus blank.

To examine whether Sch A exerts cytotoxicity in ESCC cells, we monitored the cell viability of human ESCC lines KYSE30, KYSE70, KYSE410, KYSE450, and KYSE510 after treatment with Sch A (0–80 μM) for 24 and 48 h. The cell viability, determined by the MTT assay, indicated that Sch A suppressed the viability and proliferation of ESCC cells in response to increasing concentrations and prolonged exposure (Fig. 1A–1E). After 48 hours of incubation, the IC_50_ values of Sch A were 48.84 μM, 55.90 μM, 61.82 μM, 61.78 μM, and 52.94 μM for KYSE30, KYSE70, KYSE410, KYSE450, and KYSE510 cells, respectively. In contrast, the cytotoxic effect of Sch A on human epidermal keratinocyte HEKa cells was only marginally observed (Fig. 1F). We subsequently selected KYSE30 and KYSE510 cells for further investigation, as these lines were more susceptible to Sch A treatment based on their IC_50_ values. A soft agar assay was then performed to evaluate anchorage-independent growth. Consistent with our expectations, Sch A decreased the growth of both KYSE30 and KYSE510 cells (Fig. 1G), reducing the size and number of colonies. In contrast, when treated with Ox (10 μM), colony formation was not observed in KYSE30 and KYSE510 cells (Fig. 1H and 1I).

### Sch A Induces Apoptosis of ESCC Cells by Activating JNK and p38 MAPK Signaling

**Fig. 2:**
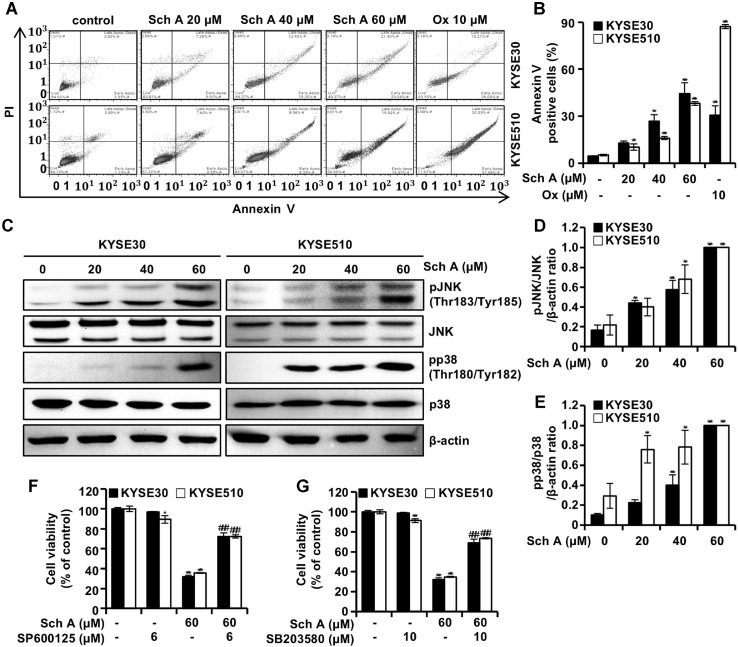
Apoptosis and activation of JNK and p38 MAPK signaling. (**A–E**) Human ESCC KYSE30 and KYSE510 cells were treated with the indicated concentrations of schisandrin A (Sch A; 0, 20, 40, and 60 μM) or oxaliplatin (Ox; 10 μM) for 48 h and analyzed by flow cytometry using annexin V/propidium iodide (PI) staining and western blotting. (**A**) Representative flow cytometry plots. (**B**) Percentage of annexin V–positive KYSE30 (closed bars) and KYSE510 cells (open bars). (**C**) Western blot analysis of p-JNK, JNK, p-p38, and p38. (**D**) Relative ratio of p-JNK/JNK protein levels normalized to β-actin. (**E**) Relative ratio of p-p38/p38. (**F, G**) Cell viability was assessed by MTT assay following Sch A treatment (60 μM) with or without pre-treatment using SP600125 (**F**) or SB203580 (**G**). Data are presented as mean ± SD (n = 3). *p < 0.05 and ***p < 0.001 versus control; ###*p* < 0.001 versus Sch A treatment alone.

Next, we examined whether Sch A induced apoptosis in ESCC KYSE30 and KYSE510 cells by conducting flow cytometry analysis using annexin V/PI double staining after 48 h of Sch A treatment (Fig. 2A and 2B). The results indicated an increase in apoptotic cells in both KYSE30 and KYSE510 cell lines. The proportion of Annexin V-positive cells (the sum of the right quadrants) rose from 4.6% to 12.9%, 26.8%, and 44.2% in KYSE30 cells, while the corresponding values for KYSE510 cells were 5.1%, 10.2%, 16%, and 38.1%. Ox treatment resulted in Annexin V-positive population of 30.6% in KYSE30 cells and 87.2% in KYSE510 cells. These results suggest that Sch A induces apoptosis in both cell lines. To investigate the involvement of JNK and p38 MAPK signaling in Sch A-induced apoptosis, we assessed protein expression levels via Western blot analysis. As shown in Fig. 2C, Sch A treatment upregulated the phosphorylation of JNK and p38 (p-JNK and p-p38) without affecting the total expression levels of these proteins. The ratios of phosphorylated to total proteins (p-JNK/JNK and p-p38/p38) increased in a dose-dependent manner following Sch A treatment (Fig. 2D and 2E). To explore the role of JNK in Sch A-induced apoptosis, KYSE30 and KYSE510 cells were pretreated with the JNK inhibitor SP600125 (6 μM) for 3 h prior to Sch A treatment (60 μM, 48 h). The reduction in cell viability induced by Sch A (31.9% in KYSE30 cells and 35.6% in KYSE510 cells) was significantly attenuated, with viability recovering to 72.3% and 72.4% in KYSE30 and KYSE510 cells, respectively. Similarly, pretreatment with the p38 MAPK inhibitor SB203580 (10 μM, 3 h) significantly reduced Sch A-induced cytotoxicity, with cell viability recovering from 32.2% and 34.8% to 68.8% and 73.6% in KYSE30 and KYSE510 cells, respectively. Treatment with SP600125 or SB203580 alone did not significantly affect cell viability.

### Sch A Induces the Generation of Reactive Oxygen Species in ESCC Cells

**Fig. 3:**
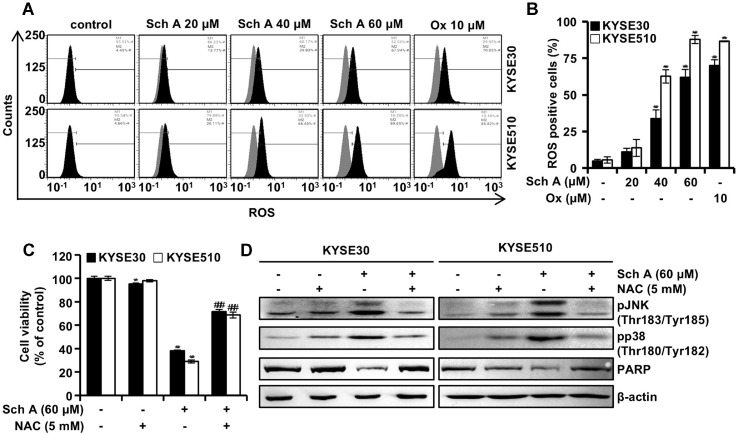
Induction of ROS generation. (**A and B**) Human ESCC KYSE30 and KYSE510 cells were treated with the indicated concentrations of schisandrin A (Sch A; 0, 20, 40, and 60 μM) or oxaliplatin (Ox; 10 μM) for 48 h and analyzed by flow cytometry using CellROX Green reagent. (**A**) Representative flow cytometry plots. (**B**) Percentage of ROS-positive KYSE30 (closed bars) and KYSE510 cells (open bars). (**C and D**) Cell viability was assessed by MTT assay following Sch A treatment (60 μM) with or without pre-treatment with N-acetylcysteine (5 mM). Data are presented as mean ± SD (n = 3). **p < 0.01 and ***p < 0.001 versus control; ###*p* < 0.001 versus Sch A treatment alone. (**D**) Western blot analysis of protein levels of p-JNK, p-p38, and PARP, with β-actin used as a loading control.

Next, we evaluated the generation of reactive oxygen species (ROS) in ESCC cells treated with Sch A by conducting flow cytometry analysis using the CellROX Green reagent after 48 h of Sch A treatment. In KYSE30 cells, the proportion of ROS-positive cells increased from 4.7% to 11.2%, 33.8%, and 62% following treatment with 20, 40, and 60 μM of Sch A, respectively. In KYSE510 cells, these values increased from 5.6% to 13.8%, 62.7%, and 87.8% (Fig. 3A and 3B). Following exposure to Ox (10 μM), the percentage of ROS-positive cells was 70.1% in KYSE30 and 86.7% in KYSE510 cells. To investigate the role of ROS induction in Sch A-induced apoptosis, KYSE30 and KYSE510 cells were pretreated with NAC (5 mM) for 3 h before receiving Sch A treatment (60 μM for 48 h). The reduction in cell viability induced by Sch A (38.1% in KYSE30 and 29% in KYSE510 cells) was significantly attenuated, with recovery to 71.6% and 68.6% in KYSE30 and KYSE510 cells, respectively (Fig. 3C). The cytotoxicity of NAC was negligible in KYSE30 cells and statistically non-significant in KYSE510 cells. Western blot analysis demonstrated that NAC pretreatment abrogated the Sch A-induced phosphorylation of JNK and p38, as well as PARP cleavage (Fig. 3D).

### Sch A Induces Cell Cycle Arrest at the G1 Phase

**Fig. 4:**
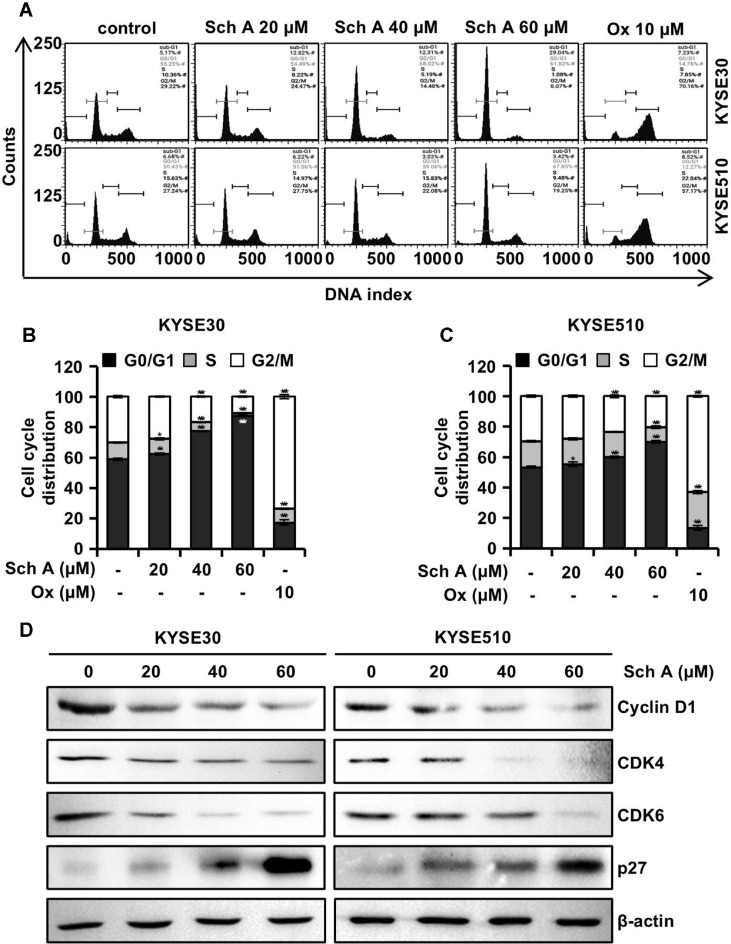
Cell cycle distribution and regulators. Human ESCC KYSE30 and KYSE510 cells were treated with the indicated concentrations of schisandrin A (Sch A; 0, 20, 40, and 60 μM) or oxaliplatin (Ox; 10 μM) for 48 h and subjected to flow cytometry analysis with PI staining and Western blot analysis. (**A**) Cell cycle flow cytometry profiles. Histogram showing cell cycle distributions of KYSE30 (**B**) and KYSE510 cells (**C**). (**D**) Western blot analysis of cyclin D1, CDK4, CDK6, and p27. β-actin was used as a loading control. Data are presented as mean ± SD (n = 3). *p < 0.05, **p < 0.01, and ***p < 0.001 versus control.

To determine the effect of Sch A on cell cycle regulation in ESCC cells, KYSE30 and KYSE510 cells were analyzed by flow cytometry using PI staining after 48 h of incubation with Sch A (20, 40, and 60 μM). In contrast, treatment with Ox (10 μM) induced a significant increase in the G2/M phase population of ESCC cells. Cell cycle distribution analysis showed that the population of KYSE30 cells in the G0/G1 phase increased from 58.9% to 62.3%, 77.3%, and 87.5% with Sch A treatment at 20, 40, and 60 μM, respectively (Fig. 4B). The corresponding values for KYSE510 cells increased from 53.3% to 55.4%, 60%, and 70.1%, respectively (Fig. 4C). Western blot analysis revealed a dose-dependent decrease in the levels of cyclin D1, CDK4, and CDK6, while p27 expression significantly increased following Sch A treatment (Fig. 4D).

### Sch A Induces Mitochondrial Dysfunction in ESCC Cells

**Fig. 5:**
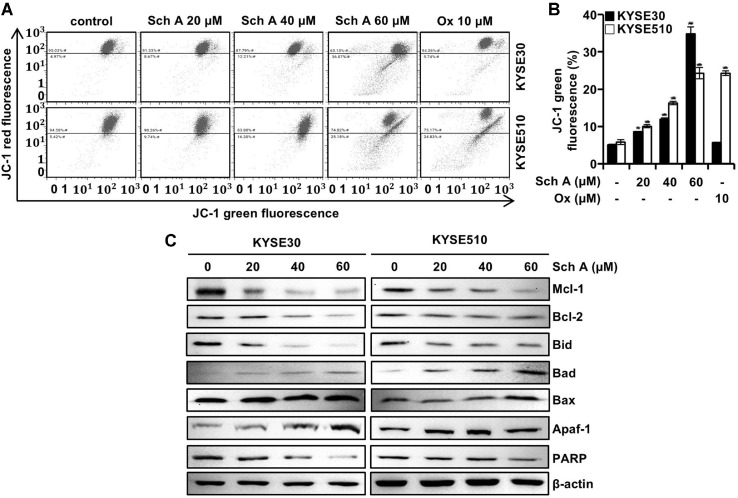
Dysregulation of MMP and Bcl2-proteins. Human ESCC KYSE30 and KYSE510 cells were treated with the indicated concentrations of schisandrin A (Sch A; 0, 20, 40, and 60 μM) or oxaliplatin (Ox; 10 μM) for 48 h and subjected to flow cytometry analysis with JC-1 staining and Western blotting. (**A and B**) Flow cytometry plots of JC-1 green staining (Left) and Percentages of JC-1 green fluorescence cells in KYSE30 (closed bars) and KYSE510 cells (open bars). (**C**) Western blot analysis of Mcl-1, Bcl-2, Bid, Bad, Bax, Apaf-1, and PARP. Data are presented as mean ± SD (n = 3). **p < 0.01 and ***p < 0.001 versus control.

To evaluate whether Sch A induces mitochondrial dysfunction in ESCC cells, we assessed mitochondrial membrane potential flow cytometry using with JC-1 staining in KYSE30 and KYSE510 cells treated with Sch A at concentrations of 20, 40, and 60 μM for 48 h. Treatment with Sch A resulted in a dose-dependent increase in the percentage of JC-1 green-positive cells, rising from 5.1% to 8.6%, 11.9%, and 34.8% in KYSE30 cells, and from 5.8% to 10.1%, 16.3%, and 24.3% in KYSE510 cells. In contrast, while treatment with Ox increased the proportion of JC-1 green-positive KYSE510 cells to 24.3%, no significant change was observed in KYSE30 cells (5.7%; Fig. 5A and 5B). Western blot analysis demonstrated a dose-dependent decrease in the expression of anti-apoptotic proteins Mcl-1 and Bcl-2, as well as the full-length form of the pro-apoptotic protein Bid. Conversely, levels of Bax, Apaf-1, and cleaved PARP increased following Sch A treatment.

### Sch A Induces Caspase Activation in ESCC Cells

**Fig. 6:**
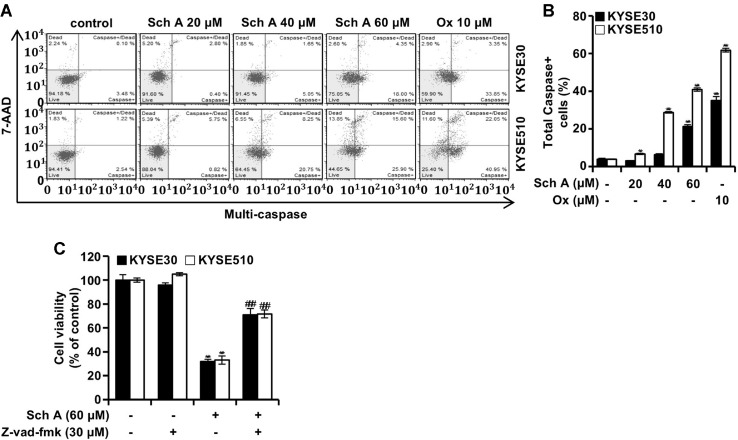
Caspase activation. Human ESCC KYSE30 and KYSE510 cells were treated with the indicated concentrations of schisandrin A (Sch A; 0, 20, 40, and 60 μM) or oxaliplatin (Ox; 10 μM) for 48 h and subjected to flow cytometry analysis using the MultiCaspase Assay Kit. (**A**) Representative flow cytometry plots. (**B**) Percentages of total caspase-positive cells in KYSE30 (closed bars) and KYSE510 cells (open bars). (**C**) Cell viability was assessed by MTT assay following Sch A treatment (60 μM) with or without pre-treatment with Z-vad-fmk (30 μM). Data are presented as mean ± SD (n = 3). **p < 0.01 and ***p < 0.001 versus control; ###*p* < 0.001.

To assess whether Sch A treatment leads to caspase activation, cells were analyzed using a multi-caspase assay kit and flow cytometry (Fig. 6A and 6B). In KYSE30 cells, the proportion of multi-caspase-positive cells increased from 3.9% to 3.0%, 6.2%, and 21.2% with Sch A treatment at 20, 40, and 60 μM, respectively. In KYSE510 cells, the corresponding values increased from 3.9% to 6.6%, 28.7%, and 40.8%. To determine if caspase activation mediates Sch A-induced cytotoxicity, ESCC cells were pretreated with Z-vad-fmk, a pan-caspase inhibitor (30 μM), for 3 h prior to treatment with Sch A (60 μM). The MTT assay indicated that cell viability, which decreased to 32% and 33.1% in KYSE30 and KYSE510 cells, respectively, after Sch A treatment, recovered to 70.9% and 71.6% with Z-vad-fmk. Z-vad-fmk alone did not significantly affect cell viability (Fig. 6C).

## Discussion

Despite the emergence of immunotherapy for ESCC, many patients remain unresponsive, highlighting the urgent need for alternative therapeutic strategies. The JNK/MAPK signaling axis serves as a critical regulatory node in this context. Dysregulation of this pathway, induced by Sch A, has been shown to effectively trigger apoptosis in prostate cancer cells [12].

Sch A exhibited cytotoxicity in all five tested ESCC cell lines, while showing only marginal cytotoxicity in HEKa cells. This indicates that the cytotoxicity is limited to cancer cells (Fig. 1A–1F). Additionally, colony formation in the two most sensitive cell lines, KYSE30 and KYSE510, was significantly reduced following Sch A treatment (Fig. 1G–1I). Although less potent, the efficacy of Sch A was comparable to that of oxaliplatin (Ox), a third-generation platinum-based anticancer agent [15], particularly in KYSE510 cells.

Induction of apoptosis is a key mechanism through which natural products exert anticancer activity [16]. Annexin V/PI double staining revealed that Sch A induced apoptosis in both KYSE30 and KYSE510 cells (Fig. 2A and 2B). Activation of JNK and p38 MAPK signaling, triggered by stress, regulates cell differentiation and death [17]. Western blot analysis showed that Sch A treatment resulted in high phosphorylation of both JNK and p38 MAPK (Fig. 2C–2E). Additionally, pretreatment with SP600125 (a JNK inhibitor) and SB203580 (a p38 MAPK inhibitor) effectively mitigated the cytotoxicity of Sch A (Fig. 2F and 2G), suggesting that both JNK and p38 MAPK mediate Sch A-induced apoptosis in ESCC cells.

Excessive levels of ROS can induce apoptosis, leading to the death of cancer cells [18, 19]. In this study, we observed that Sch A-induced ROS generation occurred in a concentration-dependent manner, reaching levels comparable to those induced by Ox treatment (Figs. 3A and 3B). NAC pretreatment confirmed that the cytotoxicity induced by Sch A was ROS-dependent (Fig. 3C). In the presence of NAC, neither JNK/p38 MAPK phosphorylation nor PARP cleavage took place, indicating that ROS generation triggers the activation of these pathways (Fig. 3D).

Perturbing the cell cycle is another effective approach to treating cancer cells [20]. Additionally, we observed an increase in the number of cells in the G0/G1 phase following Sch A treatment in both KYSE30 and KYSE510 cells (Fig. 4B and 4C). Western blot analysis revealed decreased levels of cyclin D1, CDK4, and CDK6, along with a concurrent increase in p27 (Fig. 4D), suggesting that the antiproliferative activity of Sch A is partly due to G0/G1 phase arrest. In contrast, Ox treatment resulted in a significant increase in the number of cells in the G2/M phase, indicating G2/M arrest [21].

The induction of ROS generation can disrupt mitochondrial membrane potential (MMP) [22], potentially leading to apoptosis [23]. Indeed, we observed an increase in JC-1 green–positive cells following Sch A treatment, along with changes in the levels of proteins associated with mitochondrial permeability and apoptosis (Fig. 5). The loss of MMP was accompanied by the activation of multiple caspases, a hallmark of apoptosis [24]. Furthermore, pretreatment with Z-vad-fmk confirmed that Sch A induced caspase-mediated apoptosis in KYSE30 and KYSE510 cells (Fig. 6).

In summary, we demonstrated that Sch A induces apoptosis in ESCC KYSE30 and KYSE510 cells by generating ROS, activating the JNK/p38 MAPK pathway, causing G0/G1 cell cycle arrest, inducing mitochondrial dysfunction, and activating caspases. Future studies aimed at identifying Sch A's molecular targets could help address primary resistance in ESCC.
